# Risk of incident autoimmune diseases in patients with newly diagnosed psoriatic disease: a nationwide population-based study

**DOI:** 10.1038/s41598-023-43778-4

**Published:** 2023-10-05

**Authors:** Joon Min Jung, Ye-Jee Kim, Woo Jin Lee, Chong Hyun Won, Mi Woo Lee, Sung Eun Chang

**Affiliations:** 1 Department of Dermatology, Asan Medical Center, University of Ulsan College of Medicine, 88, Olympic-ro 43-gil, Songpa-gu, Seoul, 05505 Korea; 2 Department of Clinical Epidemiology and Biostatistics, Asan Medical Center, University of Ulsan College of Medicine, Seoul, Korea

**Keywords:** Immunological disorders, Rheumatic diseases, Skin diseases

## Abstract

There are limited large population-based cohort studies on the risk of incident autoimmune diseases among patients with newly diagnosed psoriatic disease. The objective of this study was to assess the risk of autoimmune diseases in patients with newly diagnosed psoriatic disease. Using the Korean National Health Insurance Service database, patients with newly diagnosed psoriatic disease between 2007 and 2019 were included. Comparators were randomly selected and matched according to age and sex. A total of 321,354 patients with psoriatic disease and 321,354 matched comparators were included in this study. Patients with psoriatic disease had a significantly higher risk of Crohn’s disease [adjusted hazard ratio (aHR), 1.95; 95% confidence interval (CI) 1.42–2.67], ulcerative colitis (aHR, 1.65; 95% CI 1.39–1.96), systemic lupus erythematosus (aHR, 1.86; 95% CI 1.34–2.57), rheumatoid arthritis (aHR, 1.63; 95% CI 1.52–1.76), ankylosing spondylitis (aHR, 2.32; 95% CI 1.95–2.77), alopecia areata (aHR, 1.41; 95% CI 1.35–1.46), and type 1 diabetes (aHR, 1.23; 95% CI 1.11–1.37). However, the risk of Graves’ disease, Hashimoto’s disease, Sjögren’s syndrome, and systemic sclerosis was not significantly different between the groups. In conclusion, patients with newly diagnosed psoriatic disease may have a significantly increased risk of incident autoimmune diseases.

## Introduction

Psoriasis is a chronic inflammatory skin condition that affects around 3.0% of the general population^1–4^. It is characterized by well-demarcated and scaly erythematous papules or plaques on the scalp, elbows, knees, hands, feet, and trunk^4^. Psoriasis patients are negatively affected by systemic inflammation-related comorbidities such as psoriatic arthritis, and the term “psoriatic disease” often comprises both psoriasis and psoriatic arthritis.

The comorbidities of psoriatic disease have substantial clinical implications ranging from cardiovascular diseases, obesity, metabolic syndrome, type 2 diabetes, dyslipidemia, liver diseases, chronic kidney diseases, and mental health diseases to even malignancies^5–9^. Specifically, psoriasis has been independently associated with myocardial infarction^10^. The treatment of psoriatic disease may affect the cardiovascular system. Conventional systemic treatments have been associated with an increased risk of major adverse cardiovascular events^11^; however, targeted therapies have not shown an association^12^. Although defects in glucose metabolism among patients with psoriatic disease are not clearly established in previous studies, epidemiological studies suggest an association between type 2 diabetes and psoriatic disease^13,14^.

Existing evidence also indicates the autoimmune aspect of psoriatic disease^15^. Specifically, the presence of autoreactive T cells has been suggested^16^. The high expression of antimicrobial peptide LL37 has been demonstrated in the epidermis in cases of psoriasis^16,17^. LL37-specific T cells not only express cutaneous lymphocyte antigens but also produce well-known psoriatic disease-related cytokines such as INF-γ, IL-17, and IL-22^16^. Additionally, psoriasis has been shown to involve pathogenic intra-epidermal CD8^+^ T cells that recognize ADAMTS-like protein 5 on melanocytes in conjunction with HLA-C*06:02^18^. Consistent with the involvement of autoimmunity in the pathogenesis of psoriatic disease, various autoimmune diseases including inflammatory bowel disease (IBD), autoimmune thyroid disease, autoimmune rheumatic diseases, and alopecia areata have been reported to be associated with psoriatic disease^19–23^. However, the association between psoriatic disease and autoimmune diseases has not been consistently reported in previous studies^24^. In addition, the risk of these autoimmune diseases in patients with psoriatic disease could not be readily estimated in previous studies because most of them were cross-sectional in nature. Therefore, by conducting a population-based cohort study with patients with newly diagnosed psoriatic disease, we aimed to further investigate the association between psoriatic disease and autoimmune diseases as well as to estimate the risk of autoimmune diseases in patients with psoriatic disease.

## Methods

### Database

Approximately 98% of the people living in the Republic of Korea are covered by the National Health Insurance (NHI). The National Health Insurance Service (NHIS) provides information on income-based insurance type, sociodemographics, ICD-10 (International Classification of Diseases 10th Revision) code-based diagnosis, services received, and prescription records^25^. A registry for rare incurable diseases (RIDs) is part of the NHIS database. To be registered in the registry, diagnoses must be certified by physicians with predefined diagnostic criteria based on the results of comprehensive medical tests including laboratory, radiological, and histological examinations.

### Study population

The relevant institutional review board approved this study, which was conducted according to the guiding principles of the Declaration of Helsinki. To identify patients with psoriatic disease, we adopted a previously validated diagnostic algorithm for psoriatic disease in Korea^26^. Patients with psoriatic disease were defined as those who had at least one documented visit with psoriatic disease as a primary diagnostic code (psoriasis, L40; psoriatic arthritis, M071-073, and M090) and a prescription for vitamin D derivatives. The algorithm had a sensitivity of 90.8% and a specificity of 92.5%^26^. Patients with psoriatic disease were identified between January 2007 and December 2019. Patients who had any diagnostic code for psoriatic disease during the 2-year washout period from January 2005 to December 2006 were excluded to avoid prevalence-incidence bias. In addition, we excluded patients who were diagnosed with autoimmune diseases [Crohn’s disease (CD), ulcerative colitis (UC), Graves’ disease, Hashimoto’s disease, systemic lupus erythematosus (SLE), rheumatoid arthritis (RA), Sjögren’s syndrome, ankylosing spondylitis (AS), systemic sclerosis, alopecia areata, and type 1 diabetes] before being diagnosed with psoriatic disease and those who were followed up for less than 1 year. Comparators who never had a diagnostic code for psoriatic disease between January 2005 and December 2019 and who never had a diagnostic code for autoimmune diseases during the washout period were randomly extracted and matched according to age and sex at a case-to-control ratio of 1:1.

### Study design

Follow-up data for newly discovered autoimmune diseases between 2007 and 2020 were included for the cohorts. Outcomes of interest were autoimmune diseases including CD, UC, Graves’ disease, Hashimoto’s disease, SLE, RA, Sjögren’s syndrome, AS, systemic sclerosis, alopecia areata, and type 1 diabetes. To improve diagnostic accuracy, we not only used the diagnostic code but also included information on relevant drug prescription data and/or RID code for each disease in the diagnostic algorithm for outcomes of interest (Table S1). Covariates included health insurance type, Charlson Comorbidity Index (CCI)^27^, and comorbidities. Selected comorbidities were hypertension (ICD-10, I10-I13, I15), diabetes (ICD-10, E10-14), dyslipidemia (ICD-10, E78), chronic obstructive pulmonary disease (ICD-10, J44), liver cirrhosis (ICD-10, K74), chronic kidney disease (ICD-10, N18), heart failure (ICD-10, I50), and alcoholic liver disease (ICD-10, K70). Patients were considered to have a comorbidity if they had at least two visits with the corresponding diagnostic code within a year.

### Subgroup analyses

Subgroup analyses were performed according to sex and age (age < 40 years, age ≥ 40 years). Subgroup analyses according to the severity of psoriatic disease were also performed. Patients with psoriatic disease were classified as having mild psoriatic disease if they had received only topical vitamin D derivatives during the first 90 days after diagnosis. Moderate to severe psoriatic disease patients were defined as patients who had received systemic treatment or phototherapy during the first 90 days after diagnosis.

### Statistical analysis

Student *t-*test was used to compare continuous variables, and the chi-square test was used for binary and categorical variables. The incidence rates of autoimmune diseases were calculated per 100,000 person-years. By adopting a Poisson distribution, we estimated the 95% confidence interval (CIs) of the incidence rates. The risk of autoimmune diseases was determined by multivariable Cox regression models. The results are presented as hazard ratios (HRs) with 95% CIs. The number needed to harm (NNH) is defined as the number of events required to occur before one case could be attributed to the exposure and calculated^28^. All statistical tests were two-tailed. *P* values less than 0.05 were considered statistically significant. The SAS Enterprise Guide program version 7.1 (SAS Institute, Inc., Cary, NC) was used for all statistical analyses.

## Results

### Study population

**Figure 1:**
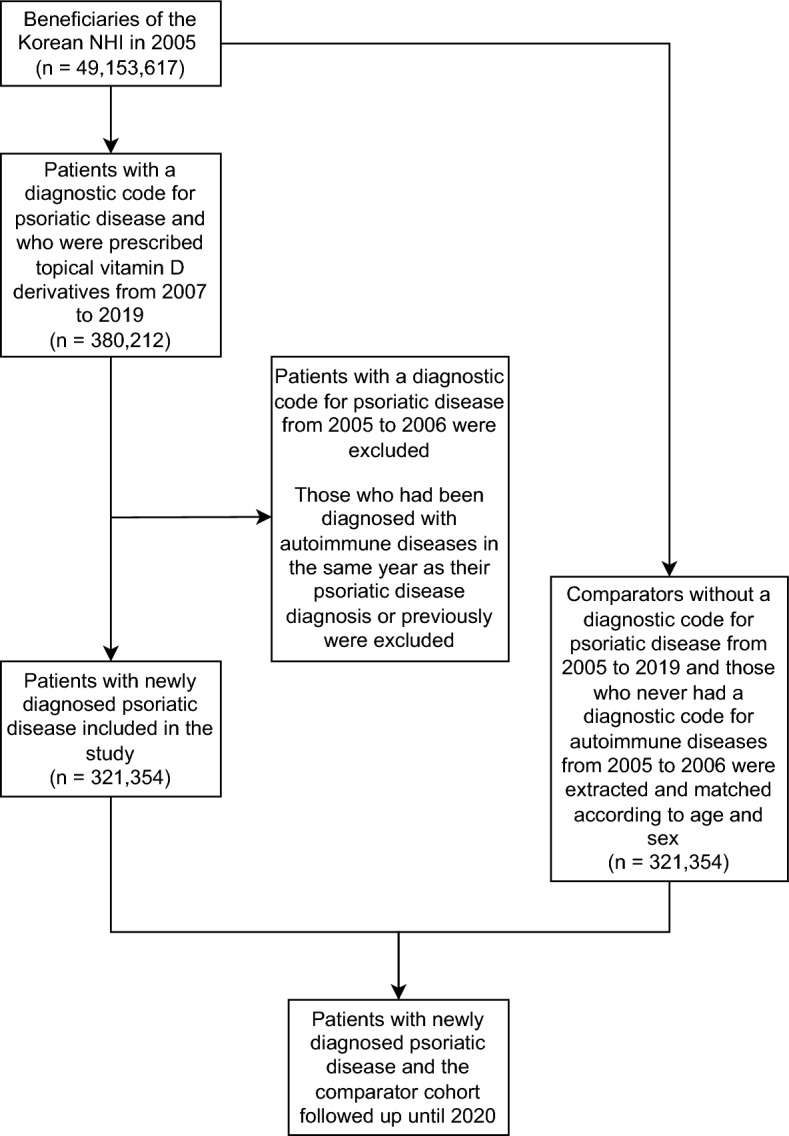
Study flow chart. *NHI* National Health Insurance.

A total of 321,354 patients with psoriatic disease and 321,354 matched comparators were included in this study (Fig. 1). The mean age was 43.05 years, and 58.9% of the subjects were males in both groups. There were more patients benefiting from medical aids among those with psoriatic disease compared with the comparators. The CCI of patients with psoriatic disease was significantly higher than that of the comparator cohort. Each comorbidity was also found to be significantly more common in patients with psoriatic disease than in the comparator cohort (Table 1).

**Table 1:** Population characteristics of matched comparators and patients with psoriatic disease.

		Matched comparators n (%)	Patients with psoriatic disease n (%)	*P* value
Total		321,354 (100)	321,354 (100)	
Sex	Male	189,352 (58.9)	189,352 (58.9)	> 0.99
	Female	132,002 (41.1)	132,002 (41.1)	
Age, years	Mean (SD)	43.05 (17.64)	43.05 (17.64)	> 0.99
	≥ 60	5,274 (1.6)	5,274 (1.6)	
	< 20	23,684 (7.4)	23,684 (7.4)	
	20–30	49,547 (15.4)	49,547 (15.4)	
	30–40	64,772 (20.2)	64,772 (20.2)	
	40–50	61,760 (19.2)	61,760 (19.2)	
	50–60	56,371 (17.5)	56,371 (17.5)	
	60–70	34,185 (10.6)	34,185 (10.6)	
	70–80	19,377 (6.0)	19,377 (6.0)	
	≥ 80	6,384 (2.0)	6,384 (2.0)	
Insurance type	Health insurance	313,059 (97.4)	308,752 (96.1)	< 0.001
	Medical aids	8,295 (2.6)	12,602 (3.9)	
Charlson Comorbidity Index	0	238,980 (74.4)	224,901 (70.0)	< 0.001
	1, 2	66,647 (20.7)	76,247 (23.7)	
	3	8,166 (2.5)	9,935 (3.1)	
	≥ 4	7,561 (2.4)	10,271 (3.2)	
Comorbidities	Hypertension	49,174 (15.3)	54,162 (16.9)	< 0.001
	Diabetes	21,721 (6.8)	25,249 (7.9)	< 0.001
	Dyslipidemia	36,021 (11.2)	42,247 (13.1)	< 0.001
	Chronic obstructive pulmonary disease	1,808 (0.6)	3,010 (0.9)	< 0.001
	Liver cirrhosis	778 (0.2)	1,272 (0.4)	< 0.001
	Chronic kidney disease	977 (0.3)	1,328 (0.4)	< 0.001
	Heart failure	2,004 (0.6)	2,652 (0.8)	< 0.001
	Alcoholic liver disease	1,953 (0.6)	3,209 (1.0)	< 0.001

*SD* standard deviation.

### Risk of autoimmune diseases in patients with psoriatic disease

**Figure 2:**
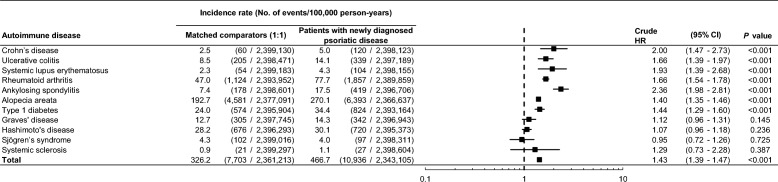
Risk of autoimmune diseases in patients with newly diagnosed psoriatic disease compared with a matched comparator cohort. *CI* confidence interval; *HR* hazard ratio.

In comparison with the matched comparator cohort, patients with psoriatic disease had a significantly higher risk of CD, UC, SLE, RA, AS, alopecia areata, and type 1 diabetes. However, the risk of Graves’ disease, Hashimoto’s disease, Sjögren’s syndrome, and systemic sclerosis was comparable between both groups (Fig. 2). The NNH for CD, UC, SLE, RA, AS, alopecia areata, and type 1 diabetes was 39,988, 17,899, 47,987, 3256, 9946, 1295, and 9567 patient-years, respectively. After adjusting for insurance type, CCI and comorbidities, the risk of CD (adjusted HR [aHR], 1.95; 95% CI 1.42–2.67), UC (aHR, 1.65; 95% CI 1.39–1.96), SLE (aHR, 1.86; 95% CI 1.34–2.57), RA (aHR, 1.63; 95% CI 1.52–1.76), AS (aHR, 2.32; 95% CI 1.95–2.77), alopecia areata (aHR, 1.41; 95% CI 1.35–1.46), and type 1 diabetes (aHR, 1.23; 95% CI 1.11–1.37) was still significantly higher in patients with psoriatic disease than in the comparator cohort.

### Risk of autoimmune diseases in patients with psoriatic disease according to sex and age

**Figure 3:**
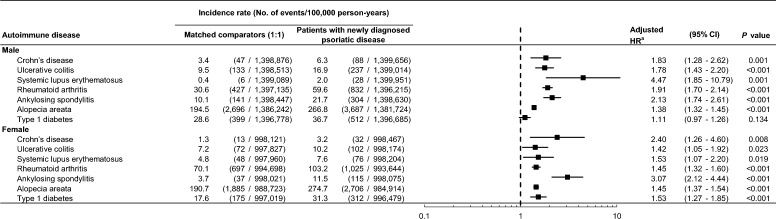
Risk of autoimmune diseases in patients with newly diagnosed psoriatic disease compared with a matched comparator cohort according to sex. *CI* confidence interval; *HR* hazard ratio. ^a^Adjusted for insurance type, Charlson Comorbidity Index, and the presence of hypertension, diabetes, dyslipidemia, chronic obstructive pulmonary disease, and heart failure.

The risk of CD, UC, SLE, RA, AS, alopecia areata, and type 1 diabetes was further analyzed according to sex and age. After adjusting for insurance type, CCI and comorbidities, all of the autoimmune diseases except for type 1 diabetes showed a significantly higher risk in patients with psoriatic disease than in the comparator cohort regardless of sex. The risk of type 1 diabetes (aHR, 1.11; 95% CI 0.97–1.26) was not significantly higher in male patients with psoriatic disease compared with male comparators after adjusting for insurance type, CCI and comorbidities (Fig. 3).

**Figure 4:**
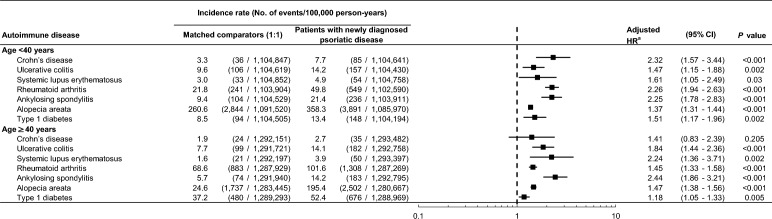
Risk of autoimmune diseases in patients with newly diagnosed psoriatic disease compared with a matched comparator cohort according to age. *CI* confidence interval; *HR* hazard ratio. ^a^Adjusted for insurance type, Charlson Comorbidity Index, and the presence of hypertension, diabetes, dyslipidemia, chronic obstructive pulmonary disease, and heart failure.

All of the autoimmune diseases except for CD showed a significantly higher risk in both younger (age < 40 years) and older (age ≥ 40 years) patients with psoriatic disease compared with their matched comparators after adjusting for insurance type, CCI and comorbidities (Fig. 4). The risk of CD (aHR, 1.41; 95% CI 0.83–2.39) was not significantly higher in older patients with psoriatic disease compared with age-matched comparators after adjusting for insurance type, CCI and comorbidities (Fig. 4).

### Risk of autoimmune diseases in patients with psoriatic disease according to the severity of the disease

Patients with mild psoriatic disease and moderate to severe psoriatic disease accounted for 79.4% (255,285/321,354) and 20.6% (66,069/321,354) of patients with psoriatic disease, respectively (Table S2). The incidence rates of CD, UC, SLE, RA, AS, alopecia areata, and type 1 diabetes were higher in both the mild and moderate to severe psoriatic disease groups than in the comparator cohort group. After adjusting for age, sex, insurance type, and CCI the risk of RA (aHR, 1.53; 95% CI 1.39–1.70), AS (aHR, 1.52; 95% CI 1.23–1.87), and type 1 diabetes (aHR, 1.22; 95% CI 1.04–1.44) was significantly higher in patients with moderate to severe psoriatic disease than in patients with mild psoriatic disease (Table S3).

## Discussion

The association between psoriatic disease and various autoimmune diseases has been suggested; however, its association may vary depending on the study protocol^19–24^. We adopted a large population-based database and more than a 10-year follow-up period to better estimate the risk of incident autoimmune diseases in patients with psoriatic disease. Furthermore, we only included incident cases of psoriatic disease because including prevalent cases may result in biased results and exaggerate the association between psoriatic disease and autoimmune diseases.

Accumulating data indicate shared pathomechanisms or genetic susceptibility loci between psoriatic disease and IBD^29^. The implications of pathogenic T helper (Th) 17 cells have been well described for both psoriatic disease and IBD^30,31^. Dysbiosis of gut microbiota has been proposed as a common pathomechanism of psoriatic disease and CD^32^. A recent Mendelian randomization study showed that genetically predicted CD was associated with a higher risk of psoriatic disease, thus supporting a causal relationship between the two diseases^33^. Our study revealed that patients with psoriatic disease had an increased risk of both CD and UC, which is consistent with previously reported cohort studies based on other databases^34,35^. In contrast to previous findings^34^, the risk of IBD was not significantly different between patients with mild psoriatic disease and those with moderate to severe psoriatic disease. In this study, the HR was higher for CD than for UC, suggesting that psoriatic disease is more strongly associated with CD than with UC. However, the NNH was lower for UC than for CD, indicating that physicians would encounter more patients with UC instead of those with CD during the follow-up of psoriatic disease patients.

Although common pathogenetic mechanisms between psoriatic disease and SLE, including the role of RUNX proteins and IL-17, have been described^36–38^, the coexistence of psoriatic disease and SLE has been reported only in some cases series^39^. In a single-center study, the prevalence of psoriatic disease was higher in patients with SLE than in the general population. However, in the study, coexisting psoriatic disease had a minimal effect on the disease activity of SLE^39^. A recent population-based study showed that the prevalence of SLE was higher in the psoriatic arthritis group than in the control group. In the study, older age and statin prescription were suggested as risk factors for SLE development^40^. The present study showed that the association between psoriatic disease and SLE was maintained regardless of age and sex. Notably, the association was stronger in the older and male patient groups. SLE development after systemic therapies for psoriatic disease, including tumor necrosis factor-α inhibitor (TNFi) therapy, has been reported^41^. However, in this study, the significant effect of systemic medications for psoriatic disease on the development of SLE was less likely considering that psoriatic disease patients who received systemic treatment did not have a higher incidence of SLE compared with that of patients who were treated with only topical anti-psoriatic medication.

The association between psoriatic disease and RA is unclear. Nevertheless, the results of some cross-sectional studies have suggested an association between psoriatic disease and RA^21,42^. In previous studies, RA patients who received TNFi therapy had a higher risk of developing psoriatic disease compared with the risk of those who received other medications^43,44^. This study showed the elevated risk of RA in patients with psoriatic disease across all age and sex groups.

Psoriasis is often considered as an extra-articular manifestation of AS. The association between psoriatic disease and AS has been mainly assessed by cross-sectional studies^45^. Cohort studies suggest that psoriasis occurs early in the disease course of AS^46,47^. Our study showed that the risk of AS was also increased in patients with newly diagnosed psoriatic disease. The association between psoriatic disease and AS observed in this study might be partially attributed to the overlapping features of AS and axial psoriatic arthritis, which may make them indistinguishable in some cases^48^. Although only a small part of the genetic overlap between AS and psoriatic arthritis is known, HLA-B*27 has been identified as a risk factor for both diseases^48^. Patients with moderate to severe psoriatic disease had a significantly higher risk of developing RA or AS compared with the risk of those with mild psoriatic disease, demonstrating the association between psoriatic disease and these diseases. However, the shared systemic medications between psoriatic disease, RA, and AS could also contribute to the stronger association between RA or AS and moderate to severe psoriatic disease as observed in this study, which would require further investigation. Notably, the NNH was lower for RA than for AS, reflecting the higher incidence of RA compared with that of AS.

A recent systematic review revealed the bilateral association between psoriatic disease and alopecia areata based on cross-sectional studies^20^. To the best of our knowledge, our study is the first to demonstrate the risk of alopecia areata in patients with psoriatic disease^20^. Despite the modest increment in the HR, the NNH was lowest for alopecia areata in patients with psoriatic disease. In addition to the common immunological background between psoriatic disease and alopecia areata^49^, systemic medications (e.g., TNFis) for psoriatic disease, which have been reported to be paradoxically associated with the development of alopecia areata, may be another link between psoriatic disease and alopecia areata^50^.

The association between type 1 diabetes and psoriatic disease has been unclear. Several studies investigating the association between diabetes and psoriatic disease did not distinguish type 1 and 2 diabetes or only included type 2 diabetes^51–53^. A previous cross-sectional study did not observe a significant association between type 1 diabetes and psoriatic disease^19^. Nevertheless, they seem to share a common pathophysiology. Both psoriatic disease and type 1 diabetes are affected by chronic inflammation-related cytokines such as tumor necrosis factor, IL-1, and IL-6^54^. In addition, Th1 and Th17 cells, driven by IL-12 and IL-23, respectively, are known to play a key role in both diseases^55^. Alefacept, initially approved for the treatment of psoriatic disease, can also prevent pancreatic beta cell destruction by targeting pathogenic T cells in type 1 diabetes^56^. Consistent with the existing theoretical background, our study showed that patients with newly diagnosed psoriatic disease had an increased risk for developing type 1 diabetes. In addition, the risk showed an increasing tendency with worsening psoriatic disease severity, demonstrating the association between psoriatic disease and type 1 diabetes.

Some studies have assessed the association between autoimmune thyroid disease and psoriatic disease, which presented conflicting results^19^. Consistent with previous case–control studies reporting that there was no difference in the prevalence of autoimmune thyroid disease between patients with psoriatic disease and controls^24,57^, the present study showed that the risk of developing autoimmune thyroid disease was not increased in patients with newly diagnosed psoriatic disease.

In previous population-based studies, the frequency of systemic sclerosis was higher in patients with psoriatic disease than in controls^19,21^. However, we did not observe any increased risk of developing systemic sclerosis in patients with newly diagnosed psoriatic disease.

This study is not without limitations. First, there may be inherent diagnostic inaccuracies as our study was based on administrative data. However, we not only used a previously validated diagnostic algorithm for psoriatic disease but also combined the diagnostic code with the prescription of medications or a highly reliable RID code for each outcome. Second, the risk of only a selected number of autoimmune diseases was assessed. Third, psoriatic disease severity was only indirectly assessed using prescription data. Fourth, although the results of the study were adjusted for insurance type, which is closely related to the economic status of each individual, other potential confounding factors including lifestyle and living environment could not be adjusted. Lastly, the generalizability of the results of our study should be examined in further studies.

In conclusion, compared with the comparator cohort, patients with newly diagnosed psoriatic disease had a higher risk of incident autoimmune diseases including CD, UC, SLE, RA, AS, alopecia areata, and type 1 diabetes. In comparison with the findings of previous studies based on prevalent cases of psoriatic disease, the results of the current study strongly indicated a shared autoimmune-related pathomechanism between psoriatic disease and autoimmune diseases. This study also demonstrated the existence of autoimmune diseases not related to psoriatic disease, such as Graves’ disease, Hashimoto’s disease, Sjögren’s syndrome, and systemic sclerosis. As the absolute risk of each psoriatic disease-related autoimmune disease assessed by the NNH was not sufficiently high, routine screening for these autoimmune diseases in patients with psoriatic disease may not be encouraged. However, considering the lowest NNH for alopecia areata and the low cost of clinical examinations, appropriate examinations may be necessary to determine the presence of comorbid alopecia areata in patients with psoriatic disease to provide individualized treatment.

### IRB approval status

The Institutional Review Board of Asan Medical Center approved this study (2021–1253). The need for informed consent from the participants was waived by the Institutional Review Board of Asan Medical Center because the data did not contain any identifiable information on individual patients. The database is strictly regulated to prevent any exposure of identifiable information.

### Supplementary Information

Supplementary Tables.

## Data Availability

All data generated or analyzed during this study are included in this published article and supplementary information file. Additional data will be provided by the corresponding author when requested.
