# Author Correction: Risk of incident autoimmune diseases in patients with newly diagnosed psoriatic disease: a nationwide population-based study

**DOI:** 10.1038/s41598-023-46498-x

**Published:** 2023-11-07

**Authors:** Joon Min Jung, Ye-Jee Kim, Woo Jin Lee, Chong Hyun Won, Mi Woo Lee, Sung Eun Chang

**Affiliations:** 1 Department of Dermatology, Asan Medical Center, University of Ulsan College of Medicine, 88, Olympic-ro 43-gil, Songpa-gu, Seoul, 05505 Korea; 2 Department of Clinical Epidemiology and Biostatistics, Asan Medical Center, University of Ulsan College of Medicine, Seoul, Korea

Correction to: *Scientific Reports* https://doi.org/10.1038/s41598-023-43778-4, published online 05 October 2023

The original version of this Article contained an error in the Funding section.

“This research was funded by grants to Chang (Grant Number 2022IL0031-1) and Jung (Grant Number 2023IL0020-1) from the Asan Institute for Life Sciences, Asan Medical Center, Seoul, Korea.”

now reads:

“This research was funded by grants to Chang (Grant Number 2022IL0031-1) and Jung (Grant Number 2023IP0123) from the Asan Institute for Life Sciences, Asan Medical Center, Seoul, Korea.”

The original Article has been corrected.

