# Supplementary material for: Risk of incident autoimmune diseases in patients with newly diagnosed psoriatic disease: a nationwide population-based study
Source: Sci Rep. 2023 Oct 5;13:16738. doi: 10.1038/s41598-023-43778-4 (PMC10556012; doi:10.1038/s41598-023-43778-4)
Supplement: Supplementary file 1 — Supplementary Tables. [file 41598_2023_43778_MOESM1_ESM.pdf]

Supplementary Information

Joon Min Jung, Ye-Jee Kim, Woo Jin Lee, Chong Hyun Won, Mi Woo Lee, Sung Eun Chang*

Risk of Incident Autoimmune Diseases in Patients with Newly Diagnosed Psoriatic Disease: A Nationwide Population-based Study

Scientific Reports

Table S1. Definitions for autoimmune diseases in the study

Table S2. Number of patients according to the severity of psoriatic disease

Table S3. Risks of autoimmune diseases in patients with moderate to severe psoriatic disease compared with those with mild psoriatic disease

This supplementary material has been provided by the authors to give readers additional information about their work.

Table S1. Definitions for autoimmune diseases in the study

Autoimmune disease	Code	Rare incurable disease code	Medications (HIRA formulary code)
Crohn's disease	K500-509	V130	-
Ulcerative colitis	K510-519	V131	-
Graves' disease	E050	-	carbimazole (4715)<br>methimazole (1918)<br>propylthiouracil (2201)
Hashimoto's disease	E063	-	levothyroxine (1836)<br>liothyronine (1844)
Systemic lupus erythematosus	M32	V136	-
Rheumatoid arthritis	M05-M06	-	adalimumab (4884)<br>cyclosporine (1392)<br>etanercept (4558)<br>infliximab (6870)<br>methotrexate (1921)<br>azathioprine (1124)<br>bucillamine (3482)<br>hydroxychloroquine (1716, 1717)<br>leflunomide (4346)<br>minocycline (1959)<br>mizoribine (1966)<br>penicillamine(d) (2099)<br>rituximab (4226)<br>sulfasalazine (2328)<br>tacrolimus (2342)
Sjögren's syndrome	M350	V139	-
Ankylosing spondylitis	M45	V140	-
Systemic sclerosis	M34	V138	-
Alopecia areata	L63		-
Type 1 diabetes	E10	-	insulin (1183, 1701, 1702, 1703, 1704, 1705, 1706, 1752, 4413, 6267, 6268, 4887, 4618, 6667, 6670,

			4849, 1753, 5074, 3278, 2156, 2157)

Abbreviations: HIRA, Health Insurance Review and Assessment Service

Table S2. Number of patients according to the severity of psoriatic disease

Disease severity	Patients with psoriatic disease (n = 321,354), n (%)
Mild	255,285 (79.4)
Moderate to severe	66,069 (20.6)
Use of systemic therapy	31,947 (9.9)
Use of photochemotherapy	32,841 (10.2)
Use of ultraviolet therapy	9,261 (2.9)

Table S3. Risks of autoimmune diseases in patients with moderate to severe psoriatic disease compared with those with mild psoriatic disease

Autoimmune disease	Mild psoriatic disease (n = 255,285)			Moderate to severe psoriatic disease (n = 66,069)			Multivariable analysis	
	Event, n	Person-years	Incidence rate (per 100,000 person-years) (95% CI)	Event, n	Person-years	Incidence rate (per 100,000 person-years) (95% CI)	Adjusted HR† (95% CI)	*P* value
Crohn's disease	100	1,888,242	5.3 (4.3–6.4)	20	509,881	3.9 (2.4–6.1)	0.73 (0.45–1.18)	0.194
Ulcerative colitis	269	1,887,542	14.3 (12.6–16.1)	70	509,647	13.7 (10.7–17.4)	0.96 (0.73–1.24)	0.729
Systemic lupus erythematosus	88	1,888,232	4.7 (3.7–5.7)	16	509,923	3.1 (1.8–5.1)	0.70 (0.41–1.20)	0.194
Rheumatoid arthritis	1,328	1,882,510	70.5 (66.8–74.4)	529	507,349	104.3 (95.6–113.5)	1.53 (1.39–1.70)	< 0.001
Ankylosing spondylitis	296	1,887,349	15.7 (13.9–17.6)	123	509,357	24.1 (20.1–28.8)	1.52 (1.23–1.87)	< 0.001
Alopecia areata	5,080	1,863,564	272.6 (265.2–280.2)	1,313	503,073	261.0 (247.1–275.5)	0.95 (0.89–1.01)	0.088
Type 1 diabetes	639	1,884,368	33.9 (31.3–36.6)	185	508,795	36.4 (31.3–42.0)	1.22 (1.04–1.44)	0.016

Abbreviations: CI, confidence interval; HR, hazard ratio

†Adjusted for age, sex, insurance type, and Charlson Comorbidity Index
